# Integrating Soft Set Theory and Fuzzy Linguistic Model to Evaluate the Performance of Training Simulation Systems

**DOI:** 10.1371/journal.pone.0162092

**Published:** 2016-09-06

**Authors:** Kuei-Hu Chang, Yung-Chia Chang, Kai Chain, Hsiang-Yu Chung

**Affiliations:** 1 Department of Management Sciences, R.O.C. Military Academy, Kaohsiung 830, Taiwan; 2 Department of Industrial Engineering and Management, National Chiao Tung University, Hsinchu 300, Taiwan; 3 Department of Computer and Information Science, R.O.C. Military Academy, Kaohsiung 830, Taiwan; Jiangnan University, CHINA

## Abstract

The advancement of high technologies and the arrival of the information age have caused changes to the modern warfare. The military forces of many countries have replaced partially real training drills with training simulation systems to achieve combat readiness. However, considerable types of training simulation systems are used in military settings. In addition, differences in system set up time, functions, the environment, and the competency of system operators, as well as incomplete information have made it difficult to evaluate the performance of training simulation systems. To address the aforementioned problems, this study integrated analytic hierarchy process, soft set theory, and the fuzzy linguistic representation model to evaluate the performance of various training simulation systems. Furthermore, importance–performance analysis was adopted to examine the influence of saving costs and training safety of training simulation systems. The findings of this study are expected to facilitate applying military training simulation systems, avoiding wasting of resources (e.g., low utility and idle time), and providing data for subsequent applications and analysis. To verify the method proposed in this study, the numerical examples of the performance evaluation of training simulation systems were adopted and compared with the numerical results of an AHP and a novel AHP-based ranking technique. The results verified that not only could expert-provided questionnaire information be fully considered to lower the repetition rate of performance ranking, but a two-dimensional graph could also be used to help administrators allocate limited resources, thereby enhancing the investment benefits and training effectiveness of a training simulation system.

## Introduction

The development of simulators can be traced back to 1929, when Edward Link developed a mechanical flight simulator that was aimed at helping new pilots familiarize with flight operating procedures. Edward’s simulator can be considered the pioneer of simulator applications. Due to technical limitations, early simulators were mostly mechanical devices, but as technologies evolve, the performance requirements of and demands for simulators have also increased. Thus, the conventional mechanical, simple-design simulators have evolved into complex, precision high-tech systems such as computers, electronics, automated control, liquid pressure system, and optical systems. Current simulators can be clustered into two categories according to usage demand and purpose. The first category involves engineering-use simulators, which are mainly used in laboratories for experimental research as well as testing, verifying, advancing, and improving existing physical products or simulator functions. The second category comprises training simulators provided for personnel training. Training simulators are characterized by various advantages; for example, using such training systems, it mitigates the risks of training casualties, saves on training costs, reduces equipment wear, facilitates autonomous learning, enhances attitude toward learning, enables exposure to battle sites, and increase training effectiveness. In recent years, advancements in technologies have engendered a diversity of simulators that can be extensively applied in many fields, such as power electronics [1], electronic applications [2], materials [3], drive security [4], traffic research [5], flight security [6], helicopter pilot training [7], performance evaluation of maritime pilots [8], aviation pilot training [9], and medical education [10]. Studies in these fields have yielded informative outcomes.

In the past, military training typically involved using actual people, vehicles, or machineries in real life. This approach easily damages the equipment used during training, shortens its lifespan, increases training cost, and potentially results in risks of accidental incidents, thereby endangering the lives and safety of trainees. Following World War II, various advanced countries have gradually incorporated simulators into their military force training because the use of simulators overcomes problems such as shortage of training equipment, poor climate (rainy or typhoon days), and adverse environmental conditions (e.g., high temperature and extreme-cold weather). In addition, simulators can be used to simulate real-life battle field environments (rainy day, snow day, thick fog, and haze). Thus, simulators have long been an integral part of national military training practices. Scholars and professional institutes in various countries have invested in experimentations and research to determine how simulators can be integrated in military training exercises. For example, research regarding pilot training [11], pilot mental workload [12, 13], and the Global Military Conflict Simulator application [14] has greatly facilitated the enhancement of military training effectiveness. However, governments worldwide have curtailed national defense budgets on a yearly basis due to global economic recession and the advocacy of pacifism (which mitigates the negative impacts of war). Such curtail indirectly affected the operation and maintenance of extant training simulators, thus impeding the execution of military mission training. Therefore, worldwide national defense departments have focused on determining how to allocate budgets under limited resources such that the usual training effectiveness and capacity are retained. However, the set up times for different types of training simulators vary, and each of these systems functions differently and is operated by people with differing backgrounds. These variable factors make it difficult to evaluate the performance of training simulators.

Because the set up times for various model training simulators differ and serve distinctive purposes, their function designs also differ completely. In other words, these systems generate both qualitative and quantitative data, and therefore, they cannot be compared and analyzed with the same standards. Proposed by Saaty [15], analytic hierarchy process (AHP) considers both qualitative and quantitative problems, performs hierarchical and structural analyses, and then quantifies the evaluated items according to questionnaire results. Subsequently, the quantified data are employed to identify the most optimal solution to the problem of interest [16]. Academic scholars have extensively applied this analytical approach to various fields. For example, Rodrigues, Ortega and Concepcion [17] combined fuzzy analytic hierarchy process and fuzzy inference system for information technology projects, considering not only the different levels of uncertainty, but also the interrelationship of risk factors. Subsequently, they demonstrated the applicability of the approach by solving actual cases of information technology projects. Rezaei, Fahim and Tavasszy [18] used fuzzy AHP to investigate supplier selection in the airline retail industry; subsequently, they applied the proposed method to an European airline and found that the method enabled selecting the most suitable supplier, thus demonstrating the applicability of the proposed method. Shen, Muduli and Brave [19] adopted AHP to evaluate the competitive priorities of improvement criteria for green supply chain management, including "appropriate implementation approach" and "continuous improvement." They determined that mining companies should focus on the improvement criteria to enhance their performance in green supply chain management, including inventory [20], and decision making management [21, 22], and other research domains. Moreover, AHP has been successfully applied in simulators such as aerospace [23], virtual environment [24].

Traditional training simulators are often evaluated through comparative analysis using numerical calculations and ranking methods. Such evaluation typically aims to attain a single goal such as how to enhance simulator performance or save on costs, and it rarely incorporates training safety into consideration. Importance–performance analysis (IPA) is an approach proposed by Martilla and James [25] to assess the service quality of corporate firms. IPA collects customer perspectives of product and service qualities and uses the collected information to elucidate the relative relationship between the importance and performance of a product or service. Subsequently, IPA analyzes this relationship based on the concept of a two-dimensional graph and then identifies the method to raise customer satisfaction and allocate service resources effectively, thereby devising the best marketing strategy. Chu and Guo [26] proposed similarity-based importance-performance analysis to assess the Tamsui Golden Riverside Bikeway, and suggested that the authorities should improve the facilities of the bikeway to make it attractive enough to support tourism activities. Chen [27] combined the IPA and Kano model to improve the deficiency in which the asymmetric and nonlinear relationships between attribute performance and customer satisfaction are ignored. Chen verified the effectiveness of the proposed method by conducting a case study of a restaurant chain. Tian, An and Gan [28] used IPA to analyze the visitors' satisfaction and competitiveness of Lotus Pond Park in China and indicated the key problems in and corresponding strategies for the development of this park to enhance and improve the overall image of the park. In recent years, IPA has been applied in studies concerning environmental protection [29], education [30], leisure and tourism [31], tourism and hospitality [32], supplier's performance [33], service quality [34], and transportation [35], providing effective marketing and business strategies for business owners.

In questionnaire survey and collection processes, incomplete questionnaires are considered invalid questionnaires; thus, some professional or valuable information may be lost, leading to inadequate consideration of the collected information. Consequently, the conclusion drawn from the collected results might deviate from the actual conditions. Molodtsov [36] proposed soft set theory, supplementing information to process the information obtained from the incomplete questionnaires so that invalid questionnaires become usable. This way, all crucial information contained in the questionnaire could be considered for without causing any loss of valuable information. Chang [37] proposed an approach that combines soft sets and hesitant fuzzy linguistic terms to solve the problem of supplier selection. Chang [37] subsequently verified that the approach can solve the problem of incomplete attribute data by effectively using a practical example involving crystal display module supplier selection. Tao et al. [38] adopted a method combining the 2-tuple linguistic terms and soft sets to solve a selection problem of investment strategy; their results demonstrated the feasibility and validity of the proposed method, and Wu [39] provides comparison of three categories of method in interval type-2 fuzzy logic systems (IT2 FLSs), and through experiments demonstrate it is not only faster than the iterative Karnik-Mendel (KM) algorithms, but also help researchers choose the most suitable structure in IT2 FLSs from computational cost perspective. Recent studies have applied this method to collate information for decision making [40–42], parameter reduction [43], risk assessment [44], stock price [45], researches on the construction of fuzzy system model [46, 47] and researches for subjective judgments [48]. In addition, concerning the consideration for and analysis of questionnaire information, Herrera and Martinez [49, 50] developed a 2-tuple fuzzy linguistic representation model, which applies 2-tuple linguistic variables to convert linguistic information into numbers. The numbers would enable full consideration of the information presented in the questionnaire, thus flexibly, reasonably reflecting the real-life situations. Zulueta et al [51] proposed a linguistic 2-tuple fusion approach for heterogeneous Environmental Impact Significance Assessment; this approach provides a flexible evaluation framework, in which experts can supply their level of knowledge and experience by using different information domains without loss of information. To ensure accuracy in the processes of assessments, Montes et al. [52] applied the 2-tuple linguistic representation model that considers both the quantitative and qualitative information of decision making in the housing market. Rao et al. [53] used a fuzzy multi-attribute group decision making technique based on a 2-tuple linguistic model to rank and select alternative City Logistics Center locations. In summary, the aforementioned methods have been applied in studying group decision making [54–56], multi-criteria decision making [57], consensus reaching processes [58], construction [59], information retrieval [60], reliability assessment [61], network security [62], and aggregation operators [63].

Chang et al. [64] proposed a training simulation performance evaluation method that integrates AHP, IPA, and 2-tuple fuzzy linguistic representation model. In this method, AHP is employed to perform a hierarchical analysis of performance evaluation problems, using pairwise comparison matrix to determine the weights of influencing factors. Subsequently, 2-tuple fuzzy linguistic representation model is adopted to fully consider the information contained in an expert questionnaire, and calculate and rank the performance scores of the training simulator. Finally, IPA is used to simultaneously consider the safety and performance scores of 10 aspects of a training simulator and then plot the values onto a two-dimensional graph. Thus, with this graph, decision-makers can clearly understand the performance of each simulator aspect and use the information as a reference for resource allocation. However, during the expert questionnaire survey process, incomplete questionnaires are deemed as invalid and are therefore disregarded. Chang et al. [64] failed to consider that crucial information might be contained within these invalid questionnaires, and thus they might have overlooked some of the information provided by the experts. As a result, the analysis results might not truly reflect the real-life conditions. Nevertheless, to address this deficiency, the present study integrated AHP, soft set theory, and the 2-tuple fuzzy linguistic representation model to evaluate the overall performance of a training simulator system. In addition, IPA was adopted to consider for both the importance and performance of a training simulator system. This study expects to provide insight into the current usage situations of training simulator systems, to avoid uneven resource distribution, which would otherwise cause a waste of training resources, and to improve existing strategies for using training simulators. The improved strategies may facilitate maximizing the benefits of limited resources, thereby effectively enhancing investment returns and training effectiveness.

The rest of this paper is organized as follows. Section 2 provides a brief literature review. A novel approach that integrates AHP, IPA and the 2-tuple fuzzy linguistic representation methods is proposed in Section 3. Section 4 presents a discussion on a numerical example of simulator benefit ranking and comparisons of the listed approaches. The final section draws on the conclusion.

## Literature Review

### Soft set theory

Soft set theory [36] was developed to resolve problems associated with data loss, incomplete data, uncertain data, and ambiguous data. Soft set is defined as follows:

F:A→P(U)

where U is the initial universal set, E is a set of parameters, and *P*(*U*) is the power set of *U*, and *A* ⊂ *E*.

**Definition 1 [44, 65].** (*F*, *A*) and (*G*, *B*) are two soft sets in a common universal set *U*, where the union of (*F*, *A*) and (*G*, *B*) is expressed by (*H*, *C*) and should satisfy the following criteria:

1. *C* = *A*∪*B*
2. ∀*e*∈*C*,

$$H(e)=\{\begin{array}{cc} F(e) & if\;e\in A-B\\ G(e) & if\;e\in B-A\\ F(e)\cup G(e) & if\;e\in A\cap B \end{array}$$ (1)

**Definition 2 [44, 65].** (*F*, *A*) and (*G*, *B*) are two soft sets in a common universal set *U*, where the intersecting set of (*F*, *A*) and (*G*, *B*) is expressed by (*H*, *C*) and should satisfy the following criteria:

1. *C* = *A*∩*B*
2. ∀*e*∈*C*, *H*(*e*) = *F*(*e*) or *G*(*e*)

**Definition 3 [65, 66].** (*F*, *A*) and (*G*, *B*) are two soft sets in a common universal set *U*; if they satisfy the following criteria, then (*F*, *A*) is the subset of (*G*, *B*), expressed as $\left(F,A)\tilde{\subset }(G,B\right)$.

1. *A* ⊆ *B*
2. ∀*e* ∈ *A*, *F*(*e*) ⊆ *G*(*e*)

**Definition 4 [65, 66].** (*F*, *A*) and (*G*, *B*) are two soft sets in a common universal set *U*; if (*F*, *A*) is the subset of (*G*, *B*) and (*G*, *B*) is the subset of (*F*, *A*), then (*F*, *A*) and (*G*, *B*) are referred to as a soft equilibrium.

### AHP method

AHP analysis is a method proposed by Professor Saaty from the University of Pittsburgh in 1980 [15]; it applies the hierarchical structure concept to decompose influencing factors of a problem into a hierarchy from high to low and then assigns a numerical value to each factor according to the relative importance of each factor, which is determined on the basis of subjective perspectives. Subsequently, a pairwise comparison matrix is developed for consistency testing to confirm the consistency of the pairwise comparison matrix. From the matrix, the weighting value of the influencing factors is derived, and finally, through numerical calculations, the influencing factors are ranked according to their importance to determine the degree of influence of each factor. AHP not only facilitates the decision making of complex problems, but also effectively simplifies the decision-making process [16]. The steps to solving a problem using an AHP analysis are described as follows.

#### (1) Defining a problem and objectives and establishing the solution hierarchy

When decision-makers apply AHP to solve problems, they must first explicate and analyze the nature of the problem of interest, identify all the factors that may influence the problem, and then summarize and classify the factors hierarchically. Subsequently, they must list the solutions of each hierarchical level in detail so that a complete hierarchy is formed.

#### (2) Calculating the weights of the influencing factors

After the hierarchy for the influencing factors of a problem is established, questionnaire survey is conducted to compare and rate the relative importance of a pair of factors, thereby completing a pairwise comparison matrix. If a problem has n influencing factors, then *n*(*n*–1)/2 pairwise comparisons in a matrix must be made. According to Saaty [15], the numerical values compared for each influencing factor in the matrix are positive (1–9), then the 9 degrees of difference of words could be sorted as equal, moderately, strongly, very strongly, and extremely, where the remaining 4 degrees are intermediate values in these 5 adjacent degrees, as shown in Table 1, and their corresponding values must exhibit a reciprocal relationship (1/2–1/9), as shown in Eq (2) [15]. Subsequently, the maximum eigenvector *λ*_max_ in Matrix *A* can be determined.

**Table 1:** The 9 degrees of difference of words definition for pairwise comparison [64].

Intensity of relative importance	Definition
1	Equal
3	Moderately
5	strongly
7	Very strongly
9	Extremely
2, 4, 6, 8	Intermediate judgment between two adjacent judgments

A=[1a12⋯a1n1a121⋯a2n⋮⋮1⋮1a1n1a2n⋯1] (2)

During the questionnaire survey, evaluations results are often inconsistent because respondents typically provide subjective responses in their questionnaires. Thus, each influencing factor exhibited a certain degree of difference when being compared. Therefore, Saaty [15] recommended replacing *n* with the maximum eigenvector in Matrix *A*, mandating that the evaluation results must be checked for consistency, or otherwise the results should be viewed as invalid.

To determine whether the pairwise comparison matrices derived from the questionnaires are consistent, consistency index (CI) must be calculated using Eq (3) and then verified using the consistency ratio (CR), as shown in Eq (4), in which random index (RI) is expressed as shown in Table 2. When the measurement results registered s CR of <0.1, the pairwise comparison matrices are consistent [67].

$$\text{CI}=\frac{\lambda_{\max}-n}{n-1}$$ (3)

$$\text{CR}=\frac{\text{CI}}{\text{RI}}$$ (4)

**Table 2:** Comparison table of RI [15].

*n*	1	2	3	4	5	6	7	8	9	10	11	12	13	14	15
RI	0.00	0.00	0.58	0.90	1.12	1.24	1.32	1.41	1.45	1.49	1.51	1.48	1.56	1.57	1.59

#### (3) Ranking the importance of each influencing factor

Next, the weighting values of each influencing factor are ranked in ascending order, and the numerical values of the problem solutions are calculated to determine the optimal solution and thereby simplify the decision-making process.

### 2-tuple fuzzy linguistic representation model

In 1965, Professor Zadeh, an US automated control expert, proposed fuzzy theory. This theory eliminates the need to use only binary logic in judging objects or events; instead, it emphasizes using fuzzy logic to describe the characteristics of objects and events in everyday life [68]. The 2-tuple fuzzy linguistic representation model was first proposed by Herrera and Martinez [50, 51]. The model is represented using the symbol (*s*,*α*), where *s* is a linguistic term and *α* is a numeric value representing the symbolic translation.

**Definition 5 [49, 50].** Let *S* = {*s*_0_,*s*_1_,…,*s*_*g*_} be a linguistic term set, and *β* ∈ [0,*g*] an aggregation of the computation result, then the following functions can be used to express *β* as 2-tuple information.

Δ:[0,g]→S×[−0.5,0.5) (5)

$$\Delta (\beta )=\{\begin{array}{cc} s_{i} & i=round(\beta )\\ \alpha =\beta -i & \alpha \in [-0.5,0.5) \end{array}$$ (6)

**Definition 6 [50, 56].** Let *x* = {(*s*_1_,*α*_1_),(*s*_2_,*α*_2_),…,(*s*_*n*_,*α*_*n*_)} be a set of 2-tuple fuzzy linguistic term, the 2-tuple arithmetic mean ${\bar{x}}^{e}$ is computed as follows.

$${\bar{x}}^{e}=\Delta \left(\sum_{j=1}^{n}\frac{1}{n}\Delta^{-1}(s_{j},\alpha_{j})\right)=\Delta \left(\frac{1}{n}\sum_{j=1}^{n}\beta_{j}\right)$$ (7)

Let (*s*_*k*_, *α*_1_) and (*s*_*l*_, *α*_2_) be two numerical values representing 2-tuple fuzzy linguistic terms, then a comparison of the size of each linguistic term is expressed as follows:

- If *k* > *l*, then (*s*_*k*_, *α*_1_) is greater than (*s*_*l*_, *α*_2_).
- If *k* = *l*, then
  1. If *α*_1_ = *α*_2,_ then (*s*_*k*_, *α*_1_) and (*s*_*l*_, *α*_2_) represent the same information.
  2. If *α*_1_ > *α*_2_, then (*s*_*k*_, *α*_1_) is greater than (*s*_*l*_, *α*_2_).
  3. If *α*_1_ < *α*_2_, then (*s*_*k*_, *α*_1_) is smaller than (*s*_*l*_, *α*_2_).

**Fig 1:**
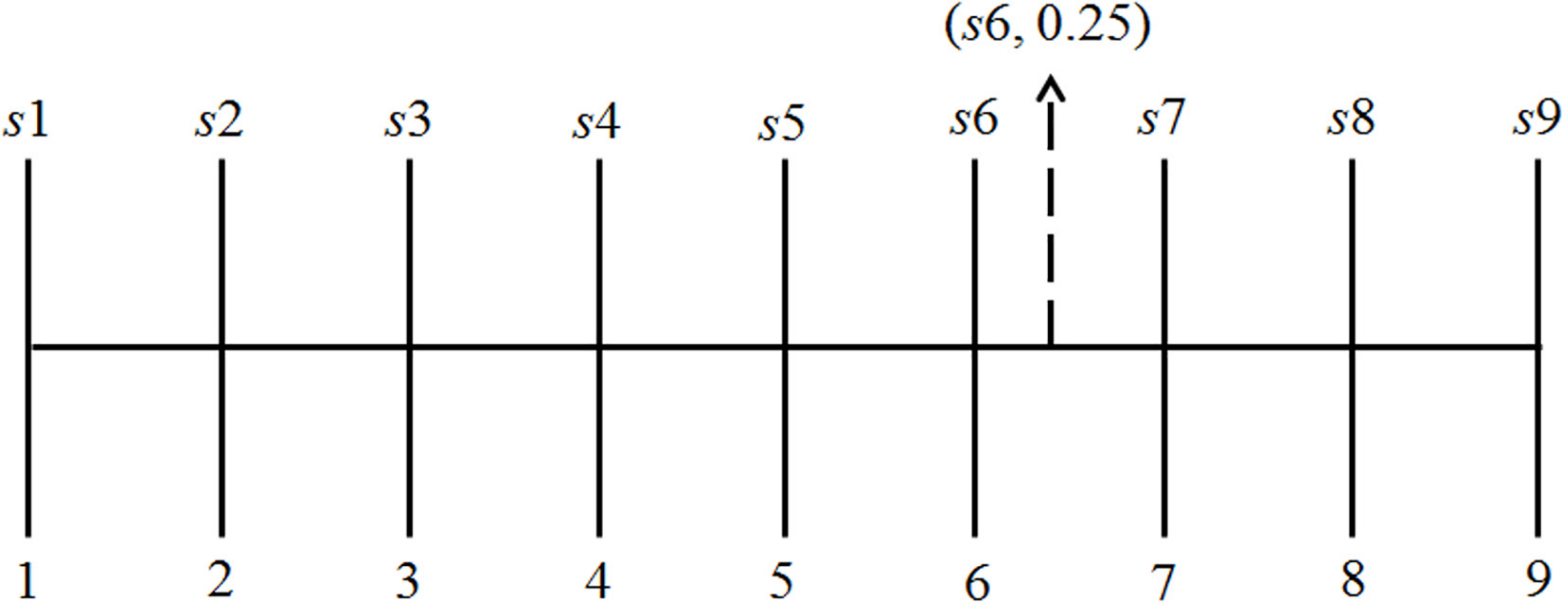
Schematic diagram of a 2-tuple fuzzy linguistic representation model.

For example, if four experts are completing a questionnaire for evaluating the performance of training simulator systems, and their ratings for a specific attribute of the training simulator are *s*5, *s*6, *s*7, and *s*7, respectively, then according to Eqs (5) and (7), the arithmetic mean is *β* = 6.25 or expressed as (*s*6, 0.25). Fig 1 illustrates the graphs of the calculation results.

### IPA method

**Fig 2:**
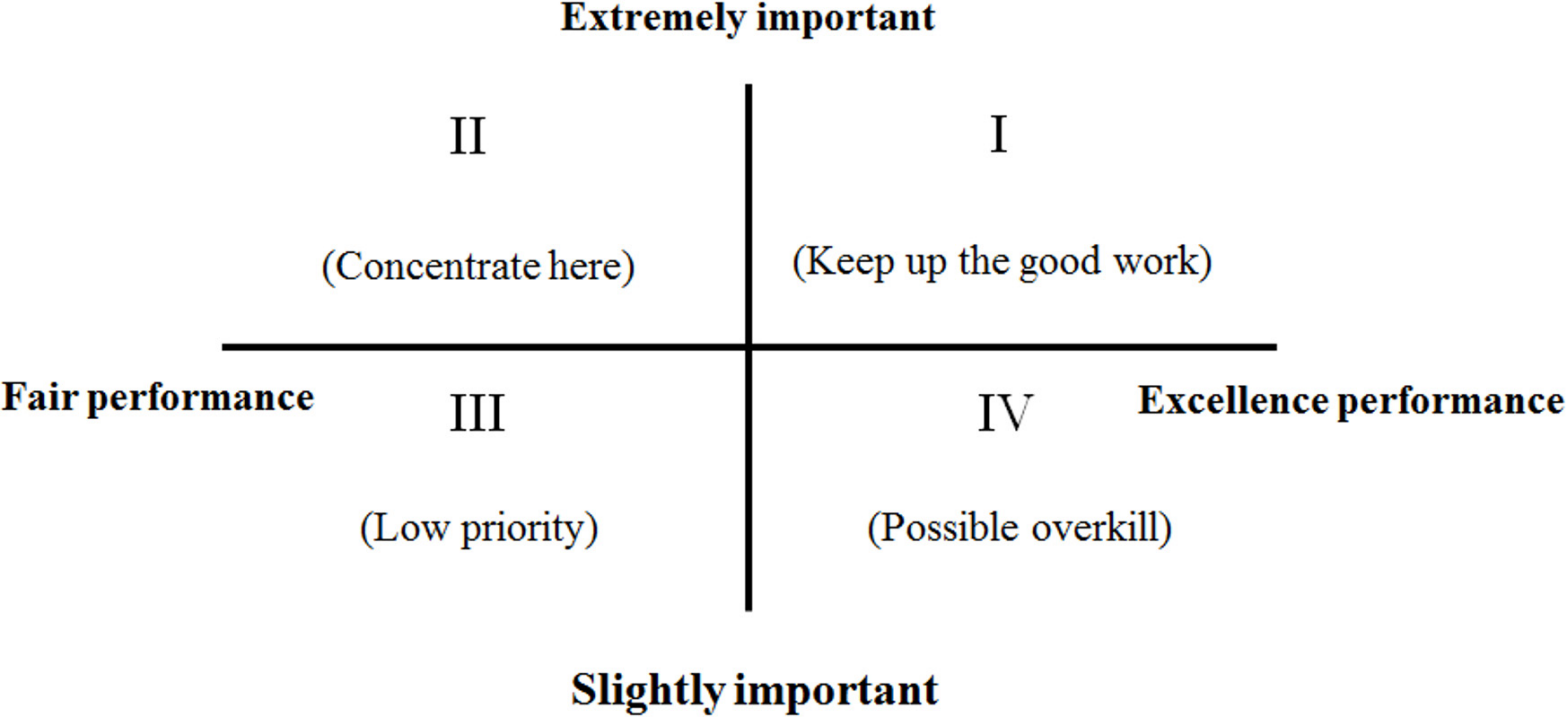
Schematic diagram showing the coordinates of an IPA scheme [25].

Developed by Martilla and James [25], the IPA method is used to investigate the weakness and strengths of attributes by using a two-dimensional graph. This method can be used to improve corporate business performance. In the two-dimensional graph, the *X* axis denotes the performance of an attribute, and the *Y* axis represents the importance of the attribute. The mean performance and importance scores are plotted into a two-dimensional graph comprising four quadrants, as shown in Fig 2.

1. Quadrant 1 (keep up the good work): Attributes in this quadrant exhibit high performance and high importance; firms should continue to maintain the competitive advantage they have in these attributes.
2. Quadrant 2 (concentrate here): Attributes in this quadrant exhibit moderate performance and high importance; firms should prioritize improving these attributes by investing in more resources.
3. Quadrant 3 (low priority): Attributes in this quadrant exhibit moderate performance and slight importance; firms do not necessary have to focus additional efforts to these attributes if they have limited resources.
4. Quadrant 4 (possible overkill): Attributes in this quadrant exhibit high performance and slight importance; firms need not overly invest in resources in these attributes.

## Proposed 2-Tuple AHP-Based Ranking Technique

**Fig 3:**
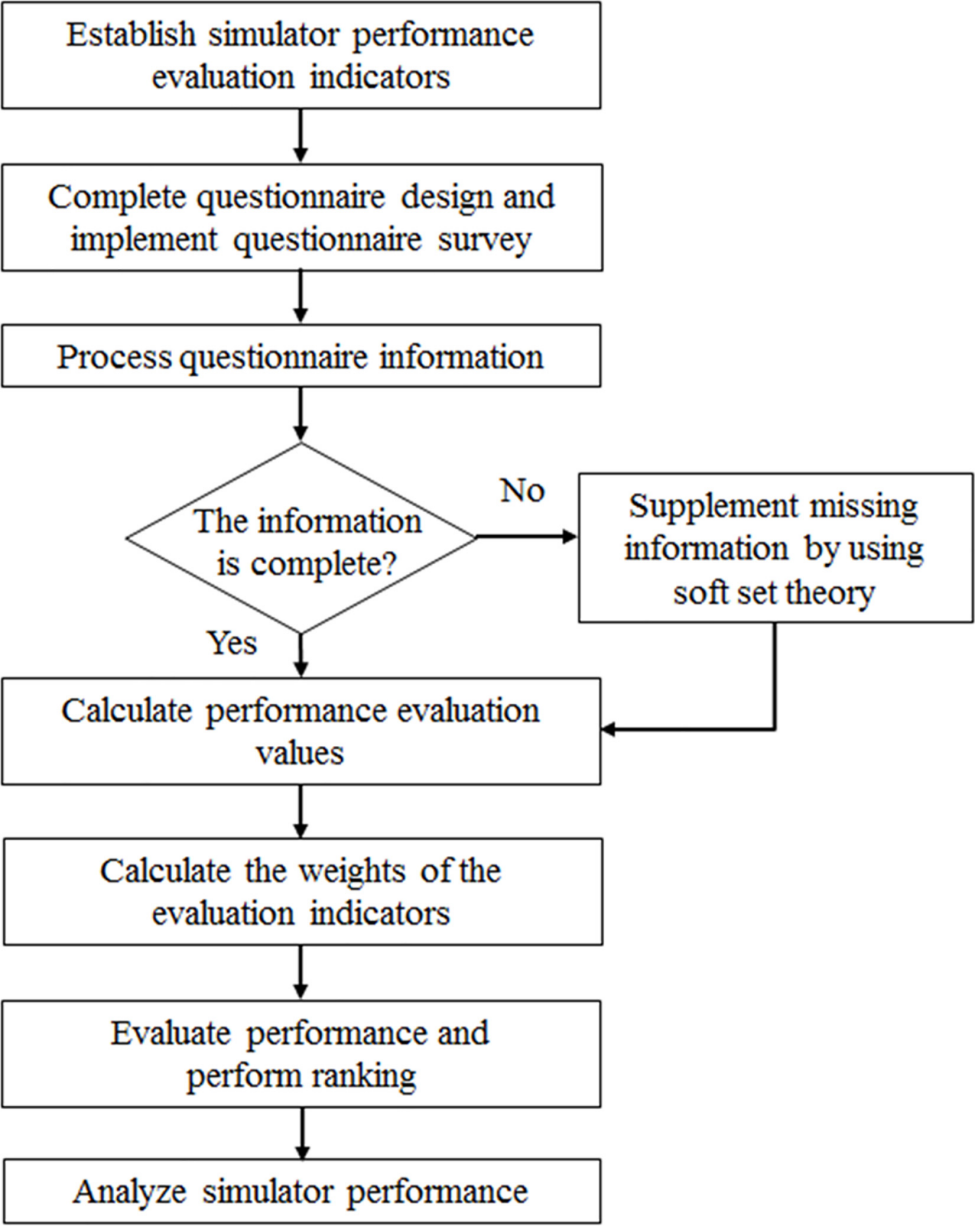
Flow diagram of the proposed method.

Although traditional AHP method can effectively consider both qualitative and quantitate problems, it can rank only the targets of a problem solution rather than simultaneously considering two solution targets. Additionally, AHP also cannot extensively consider all information presented in the questionnaire, causing the outcomes to deviate from real-life situations. To effectively address these problems, the present study proposed an evaluation method integrating 2-tuple AHP, IPA, and soft set theory. The proposed method not only fully accounts for training performance and safety, but also addresses incomplete data. Fig 3 illustrates the procedure of the proposed method.

The method proposed in this study is called “2-tuple AHP method”, which integrates soft set theory, 2-tuple AHP, and IPA comprised the following seven steps.

### Step 1. Establish simulator performance evaluation indicators

To clearly define the evaluation indicators for each simulator, AHP is applied to hierarchically analyze the problems, classify the factors that may influence the performance of a training simulator, and then establish the evaluation indicators.

### Step 2. Complete questionnaire design and implement questionnaire survey

Questionnaire is designed according to six performance evaluation indicators: Enhance teaching and training effectiveness; enhance overall training safety; effectively reduce training cost; subsequent expenses satisfy actual teaching needs; operating methods are the same as those of actual equipment; and quantity of simulators satisfies actual teaching needs. Next, the attributes of each evaluation indicator are categorized into two dimensions, Importance and Performance, in which the latter comprised enhance teaching and training effectiveness; enhance overall training safety; and effectively reduce training cost, and the former comprised subsequent expenses satisfy actual teaching needs; operating method coincides with actual equipment; and quantity of simulators satisfies actual teaching needs. Finally, a questionnaire survey is conducted.

### Step 3. Process questionnaire information

To adequately consider and apply the incomplete information of questionnaire is offered by experts, soft set theory is applied to compile the questionnaire data by supplementing information. Thereafter, complete questionnaire information is established to facilitate computing numerical values representing the performance evaluation of simulators.

### Step 4. Calculate performance evaluation values

By applying the characteristics of a 2-tuple fuzzy linguistic model, the questionnaire scores are converted into numbers that fully account for the information presented in the questionnaire. Thus, authentic expert evaluations of the simulator performance are obtained; this approach eliminates the bias in the numerical values of an evaluation caused by neglecting certain information during the computation process. Then, arithmetic means of the evaluation indicator scores, which are obtained by using the 2-tuple fuzzy linguistic model, are calculated.

### Step 5. Calculate the weights of the evaluation indicators

AHP is employed to evaluate the weights of the six evaluation indicators (i.e., enhance teaching and training effectiveness; enhance overall training safety; effectively reduce training cost; subsequent expenses satisfy actual teaching needs; operating method coincides with actual equipment; and quantity of simulators satisfies actual teaching needs), thereby completing the pairwise comparison matrix. Subsequently, consistency test is performed to ensure consistency in the evaluation results before calculating the weights of each evaluation indicator.

### Step 6. Evaluate performance and perform ranking

The calculation results obtained in Steps 4 and 5 are multiplied, and then the indicators under the evaluation dimensions (Importance and Performance) are summed to obtain the sum of the weighted average of each simulator model under the importance and performance dimensions. Thereafter, the most optimal performance ranking for each model simulator is obtained.

### Step 7. Analyze simulator performance

After ranking each model simulator according to its importance and performance in descending order, IPA is adopted, with the *X* axis denoting performance and *Y* axis denoting importance. The sum of the importance and performance evaluation scores for each model simulator is regarded as the coordinates, which are then mapped onto the two-dimensional graph. The resulting graph depicts the performance status of each model simulator and can serve as a reference for management units to allocate budgets for purchasing training simulators.

## Case Study: Training Simulator

### Overview

Present battling environments rely heavily on high-tech weapons, and considerable time and financial resources are required in training operators to master their skills in operating these high-precision weapons. However, training involving the use of actual weaponries is likely to cause personnel casualties due to negligence, which negatively affect the existing battling capability of a military force. Furthermore, the increasing awareness on human rights in recent year has prompted worldwide governments to prioritize training safety considerations. Additionally, simulators can be used indoors (free from influences of climatic conditions) and anywhere to simulate the actual situations in real-life warfare; therefore, various countries have gradually shifted toward using training simulator systems as an alternative to actual weaponries in training military armies. Nevertheless, using simulators facilitates saving training costs, reducing equipment wear and tear, preventing training accidents and casualties, enhancing overall training effectiveness, and maximizing the benefits of national defense investments.

However, due to the slow global economic development and financial constraints of countries worldwide, global national defense resources have been reduced year-by-year, limiting the budgets allocated to buying training simulators, let alone properly maintaining training simulators. These limitations consequently lead to a shortage of simulators and insufficient investment budgets, causing discrepancies in the operating methods of simulators to those of actual military equipment. Such discrepancy influences training effectiveness. Therefore, it is imperative that related national defense departments determine how to properly allocate and utilize limited resources while maintaining excellent military training capacity. To resolve the mentioned problem, the present study conducted discussion in collaboration with experts who possess more than 5 years of real-life experience in military training simulators. The discussion was aimed at defining simulator performance evaluation indicators, which are as follows: Enhance teaching and training effectiveness; enhance overall training safety; effectively reduce training cost; subsequent expenses satisfy actual teaching needs; operating methods are the same as those of actual equipment; and quantity of simulators satisfies actual teaching needs. Subsequently, these indicators were categorized into two evaluation dimensions: Importance and Performance, as shown in Table 3.

**Table 3:** Simulators benefit evaluation indicators.

Item	Evaluation indicators	Evaluation dimension
1	Effectively reduce training cost	
2	Enhance overall training safety	Importance
3	Enhance teaching and training effectiveness	
4	Subsequent expenses satisfy actual teaching needs	
5	Operating methods are the same as those of actual equipment	Performance
6	Quantity of simulators satisfies actual teaching needs	

This study conducted pairwise comparison of each evaluation indicator, assigning appropriate scores to each of the indicators, thus completing a pairwise comparison matrix. The questionnaire results are listed in Table 4. In this study, 15 military training simulators were evaluated. Concurrently, the six indicators were given ratings (1–9) according to their relative importance. Except for Experts 1 and 2 who are fully capable of rating all of the indicators because of their rich experience, the remaining Experts 3 to 10 rated the simulators according to their expertise. The evaluation results of the 15 simulators are compiled in Table 5.

**Table 4:** Comparison matrix of influencing factors.

	Evaluation items	Effectively reduce training cost	Enhance overall training safety	Enhance teaching and training effectiveness	Subsequent expenses satisfy actual teaching needs	Operating methods are the same as those of actual equipment	Quantity of simulators satisfies actual teaching needs
Effectively reduce training cost	Expert 1	1	1/6	3	3	3	2
	Expert 2	1	1/5	1	2	4	2
	Expert 3	1	1/4	1	1	5	1
	Expert 4	1	1/4	1/2	3	2	2
	Expert 5	1	1/5	1	2	2	2
	Expert 6	1	1/4	2	2	4	3
	Expert 7	1	1/2	1/2	2	3	3
	Expert 8	1	1/4	1	2	3	1/2
	Expert 9	1	1/4	1	3	4	2
	Expert 10	1	1/3	2	1	2	4
Enhance overall training safety	Expert 1		1	5	6	5	6
	Expert 2		1	4	4	6	5
	Expert 3		1	4	5	6	6
	Expert 4		1	3	7	5	7
	Expert 5		1	3	6	8	5
	Expert 6		1	3	5	6	7
	Expert 7		1	5	4	7	6
	Expert 8		1	4	5	6	5
	Expert 9		1	6	6	6	5
	Expert 10		1	5	5	5	7
Enhance teaching and training effectiveness	Expert 1			1	3	4	4
	Expert 2			1	1	5	3
	Expert 3			1	2	4	3
	Expert 4			1	2	3	2
	Expert 5			1	1/3	2	2
	Expert 6			1	1/2	1	2
	Expert 7			1	1	2	3
	Expert 8			1	3	1/2	1
	Expert 9			1	4	3	4
	Expert 10			1	3	4	3
Subsequent expenses satisfy actual teaching needs	Expert 1				1	2	2
	Expert 2				1	1	3
	Expert 3				1	3	3
	Expert 4				1	3	1/2
	Expert 5				1	1/2	1/3
	Expert 6				1	4	2
	Expert 7				1	1/2	1
	Expert 8				1	2	2
	Expert 9				1	3	3
	Expert 10				1	1	2
Operating methods are the same as those of actual equipment	Expert 1					1	1/3
	Expert 2					1	1/5
	Expert 3					1	1/4
	Expert 4					1	1/3
	Expert 5					1	1
	Expert 6					1	1
	Expert 7					1	1/2
	Expert 8					1	1/5
	Expert 9					1	1
	Expert 10					1	1/2
Quantity of simulators satisfies actual teaching needs	Expert 1						1
	Expert 2						1
	Expert 3						1
	Expert 4						1
	Expert 5						1
	Expert 6						1
	Expert 7						1
	Expert 8						1
	Expert 9						1
	Expert 10						1

**Table 5:** Expert evaluation of simulator benefit.

Benefit evaluation indicators	Simulator	A	B	C	D	E	F	G	H	I	J	K	L	M	N	O
Effectively reduce training cost	Expert 1	5	7	4	4	7	7	4	7	6	7	5	7	5	5	4
	Expert 2	6	6	4	5	6	6	3	7	5	8	5	5	6	5	4
	Expert 3	9	8	-	-	-	-	-	-	-	-	-	-	8	-	-
	Expert 4	-	-	6	6	-	-	-	-	-	-	-	-	-	-	-
	Expert 5	-	-	-	-	7	8	-	-	-	-	-	-	-	-	-
	Expert 6	-	-	-	-	-	-	4	7	7	-	-	-	-	-	-
	Expert 7	-	-	-	-	-	-	-	-	-	8	6	-	-	-	-
	Expert 8	-	-	-	-	-	-	-	-	-	-	-	6	-	-	-
	Expert 9	-	-	-	-	-	-	-	-	-	-	-	-	6	7	-
	Expert 10	-	-	-	-	-	-	-	-	-	-	-	-	-	-	5
Enhance overall training safety	Expert 1	6	6	5	4	7	6	5	7	6	7	6	5	5	6	5
	Expert 2	7	6	4	6	7	7	6	6	6	7	5	5	7	5	4
	Expert 3	8	8	-	-	-	-	-	-	-	-	-	-	6	-	-
	Expert 4	-	-	4	7	-	-	-	-	-	-	-	-	-	-	-
	Expert 5	-	-	-	-	8	7	-	-	-	-	-	-	-	-	-
	Expert 6	-	-	-	-	-	-	7	6	5	-	-	-	-	-	-
	Expert 7	-	-	-	-	-	-	-	-	-	7	5	-	-	-	-
	Expert 8	-	-	-	-	-	-	-	-	-	-	-	7	-	-	-
	Expert 9	-	-	-	-	-	-	-	-	-	-	-	-	6	7	-
	Expert 10	-	-	-	-	-	-	-	-	-	-	-	-	-	-	5
Enhance teaching and training effectiveness	Expert 1	7	5	4	7	6	6	5	6	6	8	5	5	6	6	4
	Expert 2	7	5	3	4	7	6	7	7	7	7	6	5	6	5	4
	Expert 3	8	7	-	-	-	-	-	-	-	-	-	-	7	-	-
	Expert 4	-	-	5	5	-	-	-	-	-	-	-	-	-	-	-
	Expert 5	-	-	-	-	7	8	-	-	-	-	-	-	-	-	-
	Expert 6	-	-	-	-	-	-	6	5	5	-	-	-	-	-	-
	Expert 7	-	-	-	-	-	-	-	-	-	7	6	-	-	-	-
	Expert 8	-	-	-	-	-	-	-	-	-	-	-	7	-	-	-
	Expert 9	-	-	-	-	-	-	-	-	-	-	-	-	6	6	-
	Expert 10	-	-	-	-	-	-	-	-	-	-	-	-	-	-	5
Subsequent expenses satisfy actual teaching needs	Expert 1	6	6	4	5	5	5	3	6	4	5	5	6	6	5	4
	Expert 2	7	6	3	5	5	6	2	4	5	6	4	5	5	5	4
	Expert 3	6	6	-	-	-	-	-	-	-	-	-	-	7	-	-
	Expert 4	-	-	4	6	-	-	-	-	-	-	-	-	-	-	-
	Expert 5	-	-	-	-	4	4	-	-	-	-	-	-	-	-	-
	Expert 6	-	-	-	-	-	-	4	5	4	-	-	-	-	-	-
	Expert 7	-	-	-	-	-	-	-	-	-	6	4	-	-	-	-
	Expert 8	-	-	-	-	-	-	-	-	-	-	-	5	-	-	-
	Expert 9	-	-	-	-	-	-	-	-	-	-	-	-	7	7	-
	Expert 10	-	-	-	-	-	-	-	-	-	-	-	-	-	-	4
Operating methods are the same as those of actual equipment	Expert 1	7	7	5	5	5	6	4	6	6	8	6	5	6	6	4
	Expert 2	6	7	4	6	6	5	3	6	5	7	5	5	6	5	5
	Expert 3	8	8	-	-	-	-	-	-	-	-	-	-	7	-	-
	Expert 4	-	-	4	5	-	-	-	-	-	-	-	-	-	-	-
	Expert 5	-	-	-	-	8	8	-	-	-	-	-	-	-	-	-
	Expert 6	-	-	-	-	-	-	4	6	5	-	-	-	-	-	-
	Expert 7	-	-	-	-	-	-	-	-	-	5	5	-	-	-	-
	Expert 8	-	-	-	-	-	-	-	-	-	-	-	5	-	-	-
	Expert 9	-	-	-	-	-	-	-	-	-	-	-	-	5	7	-
	Expert 10	-	-	-	-	-	-	-	-	-	-	-	-	-	-	3
Quantity of simulators satisfies actual teaching needs	Expert 1	5	6	4	5	6	6	3	6	5	6	5	6	4	4	5
	Expert 2	6	6	6	4	6	5	4	5	4	5	5	4	4	5	5
	Expert 3	7	7	-	-	-	-	-	-	-	-	-	-	6	-	-
	Expert 4	-	-	5	6	-	-	-	-	-	-	-	-	-	-	-
	Expert 5	-	-	-	-	7	6	-	-	-	-	-	-	-	-	-
	Expert 6	-	-	-	-	-	-	3	7	6	-	-	-	-	-	-
	Expert 7	-	-	-	-	-	-	-	-	-	6	4	-	-	-	-
	Expert 8	-	-	-	-	-	-	-	-	-	-	-	5	-	-	-
	Expert 9	-	-	-	-	-	-	-	-	-	-	-	-	6	6	-
	Expert 10	-	-	-	-	-	-	-	-	-	-	-	-	-	-	4

### Solution based on the traditional AHP method

#### Weighting calculation

To calculate the weighting values of the simulator performance evaluation indicators by using AHP, the simulator ratings (Table 4) provided by each expert for the evaluation indicators were computed to obtain the arithmetic means. After the standardized matrix of the relative weighting is produced, as shown in Table 6, weighting calculation was performed on each evaluation indicator.

**Table 6:** Pairwise comparison matrix of influencing factors.

Evaluating factors	Effectively reduce training cost	Enhance overall training safety	Enhance teaching and training effectiveness	Subsequent expenses satisfy actual teaching needs	Operating methods are the same as those of actual equipment	Quantity of simulators satisfies actual teaching needs
Effectively reduce training cost	—	1/4	1	2	3	2
Enhance overall training safety	4	—	4	5	6	6
Enhance teaching and training effectiveness	1	1/4	—	2	3	3
Subsequent expenses satisfy actual teaching needs	1/2	1/5	1/2	—	2	2
Operating methods are the same as those of actual equipment	1/3	1/6	1/3	1/2	—	1/2
Quantity of simulators satisfies actual teaching needs	1/2	1/6	1/3	1/2	2	—

As outlined in Section 2.2, consistency testing must be performed on the weighting values of the evaluation indicators of the evaluated simulators. Specifically, the weighting is verified as consistent when CR is less than 0.1. The present study adopted AHP problem-solving software (Expert Choice 2000) to calculate the weights of each evaluation indicator. The calculation results revealed a CR of 0.02, conforming to the consistency standard. In addition, the evaluation indicators under the importance dimension were examined according to their weightings, and the results showed that “enhance overall training safety” was rated as being the most important with a weighting value of 0.475, following by “enhance teaching and training effectiveness (0.160), and “effectively reduce training cost” (0.149).

Concerning the performance measures of the simulator evaluation, “subsequent expenses satisfy actual teaching needs” registered the heaviest weight at 0.096, followed by the “quantity of simulators satisfies actual teaching needs” (0.070) and “operating methods are the same as those of actual equipment” (0.051).

#### Simulator benefit analysis

This section provides a discussion of the results presented in Table 5 regarding the expert ratings of the simulator performance. However, because only Experts 1 and 2 completed their questionnaire fully, the other questionnaires (completed by Experts 3 to 10) were considered invalid because they were incomplete. Therefore, only the questionnaires completed by Experts 1 and 2 were subjected to arithmetic mean calculation (rounded off to the nearest ten), the results of which are presented in Table 7. Next, the weighting values of each evaluation indicator were multiplied with its corresponding ratings (shown in Table 7). The obtained weighted average and ranking are tabulated in Table 8.

**Table 7:** Ratings of simulator evaluation indicators.

			Importance			Performance	
	Benefit indicators	Effectively reduce training cost	Enhance overall training safety	Enhance teaching and training effectiveness	Subsequent expenses satisfy actual teaching needs	Operating methods are the same as those of actual equipment	Quantity of simulators satisfies actual teaching needs
1	Simulator A	6	7	7	7	7	6
2	Simulator B	7	6	6	6	7	6
3	Simulator C	4	5	4	4	5	5
4	Simulator D	5	5	6	5	6	5
5	Simulator E	7	7	7	5	6	6
6	Simulator F	7	7	6	6	6	6
7	Simulator G	4	6	6	3	4	5
8	Simulator H	7	7	7	5	6	6
9	Simulator I	6	6	7	5	6	5
10	Simulator J	8	7	8	6	8	6
11	Simulator K	5	6	6	5	6	5
12	Simulator L	6	5	5	6	5	5
13	Simulator M	6	6	6	6	5	4
14	Simulator N	5	6	6	5	6	5
15	Simulator O	4	5	4	4	5	5

**Table 8:** Weighted average and ranking of the simulator evaluation indicators.

			Importance			Performance		Total
	Benefit indicators	Effectively reduce training cost	Enhance overall training safety	Enhance teaching and training effectiveness	Subsequent expenses satisfy actual teaching needs	Operating methods are the same as those of actual equipment	Quantity of simulators satisfies actual teaching needs	
1	Simulator A	0.89	3.33	1.12	0.67	0.36	0.42	6.79
2	Simulator B	1.04	2.85	0.96	0.58	0.36	0.42	6.21
3	Simulator C	0.60	2.38	0.64	0.38	0.26	0.35	4.60
4	Simulator D	0.75	2.38	0.96	0.48	0.31	0.35	5.22
5	Simulator E	1.04	3.33	1.12	0.48	0.31	0.42	6.69
6	Simulator F	1.04	3.33	0.96	0.58	0.31	0.42	6.63
7	Simulator G	0.60	2.85	0.96	0.29	0.20	0.35	5.25
8	Simulator H	1.04	3.33	1.12	0.48	0.31	0.42	6.69
9	Simulator I	0.89	2.85	1.12	0.48	0.31	0.35	6.00
10	Simulator J	1.19	3.33	1.28	0.58	0.41	0.42	7.20
11	Simulator K	0.75	2.85	0.96	0.48	0.31	0.35	5.69
12	Simulator L	0.89	2.38	0.80	0.58	0.26	0.35	5.25
13	Simulator M	0.89	2.85	0.96	0.58	0.26	0.28	5.82
14	Simulator N	0.75	2.85	0.96	0.48	0.31	0.35	5.69
15	Simulator O	0.60	2.38	0.64	0.38	0.26	0.35	4.60

### Solution based on the AHP-based proposed method proposed by Chang et al. [64]

#### Weighting calculation

Chang et al. [64] integrated AHP, 2-tuple fuzzy linguistic representation model, and IPA to evaluate simulator performance. When calculating the weighting of each simulator evaluation indicator, they also applied AHP, with calculation methods being the same as those described in Section 4.2.1.

#### Obtaining the performance evaluation scores

Because only Experts 1 and 2 completed their questionnaire fully, only their questionnaires were calculated for arithmetic means. For Simulator A, Experts 1 and 2 respectively gave this simulator a rating of 6 and 5, the arithmetic mean of which is “5.5”. To fully account for the information provided in the questionnaire, Chang et al. [64] applied the 2-tuple fuzzy linguistic model to convert the linguistic information into numerical values (e.g., [*s*6,-0.5]), and adopted the same method for converting the linguistic information of other items. In the present study, the values obtained after conversion are tabulated in Table 9, and the values were then multiplied by the weighting values of the evaluation indicators from section 4.2.1, yielding results as shown in Table 10.

**Table 9:** Ratings of the simulator evaluation indicators.

			Importance			Performance	
	Benefit indicators	Effectively reduce training cost	Enhance overall training safety	Enhance teaching and training effectiveness	Subsequent expenses satisfy actual teaching needs	Operating methods are the same as those of actual equipment	Quantity of simulators satisfies actual teaching needs
1	Simulator A	(*s*6,-0.5)	(*s*7,-0.5)	(*s*7,0)	(*s*7,-0.5)	(*s*7,-0.5)	(*s*6,0)
2	Simulator B	(*s*7,0)	(*s*6,0)	(*s*6,0)	(*s*6,0)	(*s*7,0)	(*s*6,0)
3	Simulator C	(*s*4,0)	(*s*5,-0.5)	(*s*4,-0.5)	(*s*4,-0.5)	(*s*5,-0.5)	(*s*5,0)
4	Simulator D	(*s*5,-0.5)	(*s*5,0)	(*s*6,-0.5)	(*s*5,0)	(*s*6,-0.5)	(*s*5,-0.5)
5	Simulator E	(*s*7,-0.5)	(*s*7,0)	(*s*7,-0.5)	(*s*5,0)	(*s*6,-0.5)	(*s*6,0)
6	Simulator F	(*s*7,-0.5)	(*s*7,-0.5)	(*s*6,0)	(*s*6,-0.5)	(*s*6,-0.5)	(*s*6,-0.5)
7	Simulator G	(*s*4,-0.5)	(*s*6,-0.5)	(*s*6,0)	(*s*3,-0.5)	(*s*4,-0.5)	(*s*5,-0.5)
8	Simulator H	(*s*7,0)	(*s*7,-0.5)	(*s*7,-0.5)	(*s*5,0)	(*s*6,0)	(*s*6,-0.5)
9	Simulator I	(*s*6,-0.5)	(*s*6,0)	(*s*7,-0.5)	(*s*5,-0.5)	(*s*6,-0.5)	(*s*5,-0.5)
10	Simulator J	(*s*8,-0.5)	(*s*7,0)	(*s*8,-0.5)	(*s*6,-0.5)	(*s*8,-0.5)	(*s*6,-0.5)
11	Simulator K	(*s*5,0)	(*s*6,-0.5)	(*s*6,-0.5)	(*s*5,-0.5)	(*s*6,-0.5)	(*s*5,0)
12	Simulator L	(*s*6,0)	(*s*5,0)	(*s*5,0)	(*s*6,-0.5)	(*s*5,0)	(*s*5,0)
13	Simulator M	(*s*6,-0.5)	(*s*6,0)	(*s*6,0)	(*s*6,-0.5)	(*s*6,0)	(*s*4,0)
14	Simulator N	(*s*5,0)	(*s*6,-0.5)	(*s*6,-0.5)	(*s*5,0)	(*s*6,-0.5)	(*s*5,-0.5)
15	Simulator O	(*s*4,0)	(*s*5,-0.5)	(*s*4,0)	(*s*4,0)	(*s*5,-0.5)	(*s*5,0)

**Table 10:** Weighted average of the simulator evaluation indicators.

			Importance			Performance	
	Benefit indicators	Effectively reduce training cost	Enhance overall training safety	Enhance teaching and training effectiveness	Subsequent expenses satisfy actual teaching needs	Operating methods are the same as those of actual equipment	Quantity of simulators satisfies actual teaching needs
1	Simulator A	(*s*1,-0.18)	(*s*3,0.09)	(*s*1,0.12)	(*s*1,-0.38)	(*s*0,0.33)	(*s*0,0.42)
2	Simulator B	(*s*1,0.04)	(*s*3,-0.15)	(*s*1,-0.04)	(*s*1,-0.42)	(*s*0,0.36)	(*s*0,0.42)
3	Simulator C	(*s*1,-0.40)	(*s*2,0.14)	(*s*1,-0.44)	(*s*0,0.34)	(*s*0,0.23)	(*s*0,0.35)
4	Simulator D	(*s*1,-0.33)	(*s*2,0.38)	(*s*1,-0.12)	(*s*0,0.48)	(*s*0,0.28)	(*s*0,0.32)
5	Simulator E	(*s*1,-0.03)	(*s*3,0.33)	(*s*1,0.04)	(*s*0,0.48)	(*s*0,0.28)	(*s*0,0.42)
6	Simulator F	(*s*1,-0.03)	(*s*3,0.09)	(*s*1,-0.04)	(*s*1,-0.47)	(*s*0,0.28)	(*s*0,0.39)
7	Simulator G	(*s*1,-0.48)	(*s*3,-0.39)	(*s*1,-0.04)	(*s*0,0.24)	(*s*0,0.18)	(*s*0,0.32)
8	Simulator H	(*s*1,0.04)	(*s*3,0.09)	(*s*1,0.04)	(*s*0,0.48)	(*s*0,0.31)	(*s*0,0.39)
9	Simulator I	(*s*1,-0.18)	(*s*3,-0.15)	(*s*1,0.04)	(*s*0,0.43)	(*s*0,0.28)	(*s*0,0.32)
10	Simulator J	(*s*1,0.12)	(*s*3,0.33)	(*s*1,0.20)	(*s*1,-0.47)	(*s*0,0.38)	(*s*0,0.39)
11	Simulator K	(*s*1,-0.25)	(*s*3,-0.39)	(*s*1,-0.12)	(*s*0,0.43)	(*s*0,0.28)	(*s*0,0.35)
12	Simulator L	(*s*1,-0.11)	(*s*2,0.38)	(*s*1,-0.20)	(*s*1,-0.47)	(*s*0,0.26)	(*s*0,0.35)
13	Simulator M	(*s*1,-0.18)	(*s*3,-0.15)	(*s*1,-0.04)	(*s*1,-0.47)	(*s*0,0.31)	(*s*0,0.28)
14	Simulator N	(*s*1,-0.25)	(*s*3,-0.39)	(*s*1,-0.12)	(*s*0,0.48)	(*s*0,0.28)	(*s*0,0.32)
15	Simulator O	(*s*1,-0.40)	(*s*2,0.14)	(*s*1,-0.36)	(*s*0,0.38)	(*s*0,0.23)	(*s*0,0.35)

#### Simulator performance analysis

**Fig 4:**
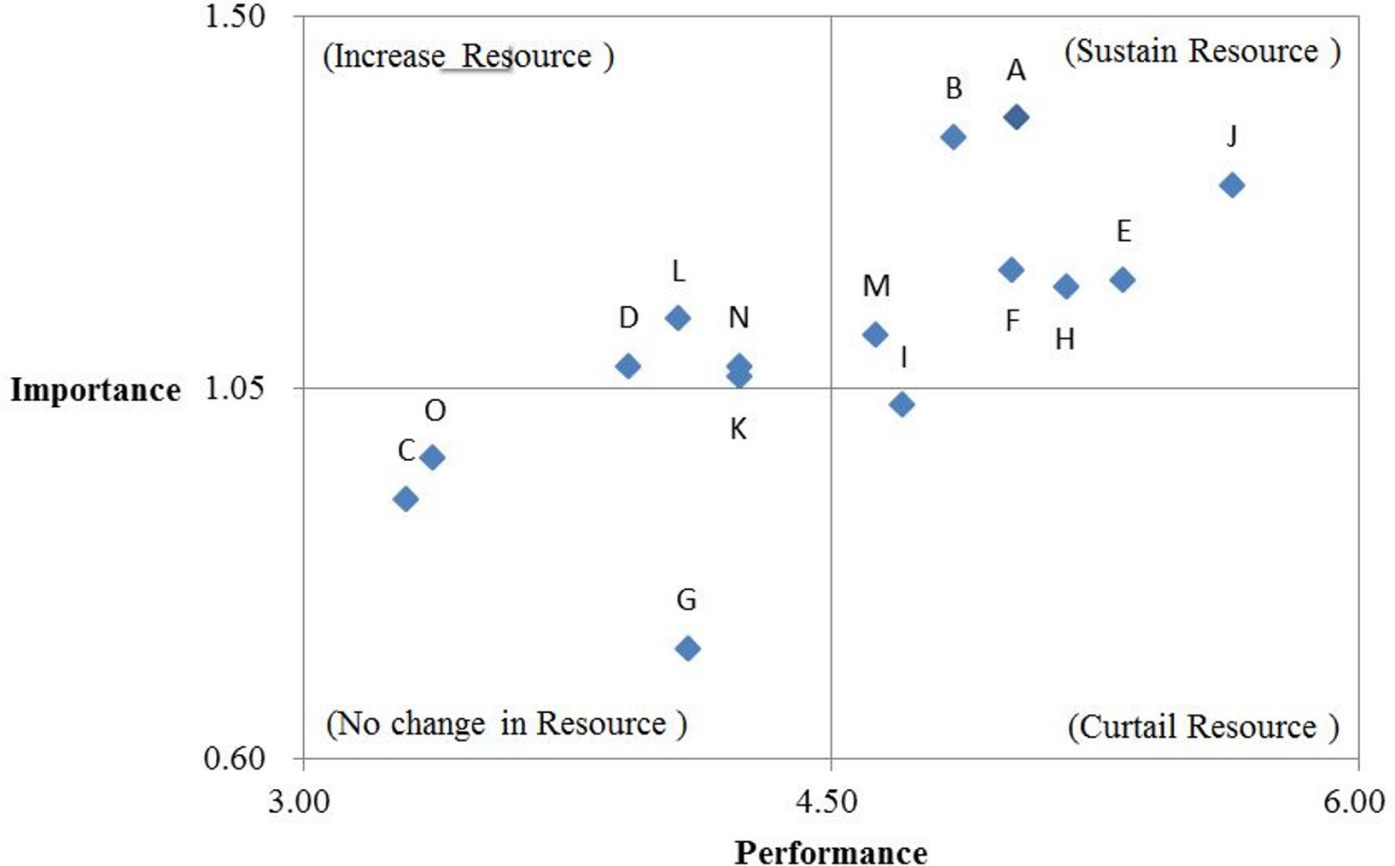
IPA diagram.

The six evaluation indicators, listed in Table 10, were separately summed and ranked according to their respective importance and performance, before the overall performance ranking of each evaluated simulator was performed. As shown in Table 11, IPA was adopted to analyze the performance of 15 simulators; “performance” was employed as the *X* axis on the two-dimensional quadrant diagram, and “importance” was used as the *Y* axis. The mean value of the importance and performance values shown in Table 11 was calculated as (4.50, 1.05), which was used as the central point of the *X* and *Y* axes. Subsequently, the upper and lower limits of the *X* and *Y* axes were identified from among the distribution of numerical values, plotting the performance value of a simulator onto the two-dimensional quadrant diagram (Fig 4). The four quadrants are explained as follows.

**Table 11:** Rankings of the simulator evaluation indicators.

		Importance		Performance		Total
	Benefit Indicators	Aggregated value of weight average	Sorting of weight average	Aggregated value of weight average	Sorting of weight average	
1	Simulator A	(*s*5,0.03)	4	(*s*1,0.38)	1	(*s*6,0.40)
2	Simulator B	(*s*5,-0.15)	6	(*s*1,0.35)	2	(*s*6,0.21)
3	Simulator C	(*s*3,0.29)	15	(*s*1,-0.08)	14	(*s*4,0.21)
4	Simulator D	(*s*4,-0.07)	13	(*s*1,0.08)	9	(*s*5,0)
5	Simulator E	(*s*5,0.33)	2	(*s*1,0.18)	5	(*s*7,-0.49)
6	Simulator F	(*s*5,0.02)	5	(*s*1,0.19)	4	(*s*6,0.21)
7	Simulator G	(*s*4,0.09)	11	(*s*1,-0.27)	15	(*s*5,-0.17)
8	Simulator H	(*s*5,0.17)	3	(*s*1,0.17)	6	(*s*6,0.34)
9	Simulator I	(*s*5,-0.29)	7	(*s*1,0.03)	12	(*s*6,-0.26)
10	Simulator J	(*s*6,-0.36)	1	(*s*1,0.30)	3	(*s*7,-0.06)
11	Simulator K	(*s*4,0.24)	9	(*s*1,0.06)	11	(*s*5,0.30)
12	Simulator L	(*s*4,0.07)	12	(*s*1,0.13)	7	(*s*5,0.20)
13	Simulator M	(*s*5,-0.37)	8	(*s*1,0.11)	8	(*s*6,-0.26)
14	Simulator N	(*s*4,0.24)	9	(*s*1,0.08)	9	(*s*5,0.31)
15	Simulator O	(s3,0.37)	14	(*s*1,-0.04)	13	(*s*4,0.34)

1. Quadrant I (sustain resources): This quadrant comprised Simulators A, B, E, F, H, J, and M, indicating that these simulators significantly enhanced training safety and effectiveness, and also exhibited better performance. Therefore, these simulators should be considered as top priority for continuous maintenance.
2. Quadrant II (increase resources): This quadrant comprised Simulators D, K, L, and N, suggesting that these simulators effectively enhanced original equipment training safety and effectiveness. However, the experts believed that the performance of these simulators require further improvement. Thus, management units should strengthen resource allocations for improving the performance of simulators in this quadrant.
3. Quadrant III (no change in resources): This quadrant comprised Simulators C, G, and O, indicating that the importance and performance of these simulators are incomparable to those in the other quadrants. Therefore, overinvestment in these simulators is not necessary to prevent wasting resources.
4. Quadrant IV (curtail resources): This quadrant comprised only Simulator I, suggesting that the performance of this simulator is recognized by the experts. However, this simulator is not as important as other simulators, and thus resource adjustment for this simulator can be considered to maximize the overall benefits of resource utilization.

### Solution based on the proposed method

#### Steps 1 and 2: Establish evaluation indicators, complete questionnaire design, and implement questionnaire survey

The proposed method integrated soft set theory, 2-tuple AHP, and IPA, applying their respective skills in solving problems to evaluate the performance of various simulators. First, Steps 1 and 2 (outlined in Section 3) were followed to establish the simulator performance evaluation indicators, design the questionnaires, and conduct the questionnaire survey.

#### Step 3: Process questionnaire information

This study adopted the traditional questionnaire analysis approach. Except for Experts 1 and 2’s questionnaires that contained complete information, the remaining questionnaires were incomplete and thus were regarded as invalid questionnaires. To fully account for the experts’ ratings, the proposed method adopted the soft set theory to supplement the incomplete information and summarize the information. For Simulator A, Experts 1, 2, and 3 rated the indicator, “effectively reduce training cost,” as 5, 6, and 9, respectively. These three ratings were calculated for arithmetic mean, yielding 6.67, which was filled in the rating columns of Experts 4 to 10. The missing information for the remaining items was completed in reference to Simulator A’s information, thus completing the questionnaire information.

#### Step 4: Calculate performance evaluation values

The results obtained from Step 3 were summarized and compiled and then subjected to 2-tuple fuzzy linguistic representation model to convert the linguistic information into numerical values. For simulator A, the arithmetic mean of which is “6.67”. To fully account for the information provided in the questionnaire, the proposed method according to Eqs (5) and (6) to converts the linguistic information into numerical values (e.g., [s7,-0.23]), and adopted the same method for converting the linguistic information of other items. As shown in Table 12, fully considering the questionnaire information avoided the slight bias in the numerical values during the calculation process. Consequently, the performance values for each simulator could be calculated more precisely.

**Table 12:** Simulator evaluation indicators.

			Importance			Performance	
	Benefit indicators	Effectively reduce training cost	Enhance overall training safety	Enhance teaching and training effectiveness	Subsequent expenses satisfy actual teaching needs	Operating methods are the same as those of actual equipment	Quantity of simulators satisfies actual teaching needs
1	Simulator A	(*s*7,-0.23)	(*s*7,0)	(*s*7,0.03)	(*s*6,0.33)	(*s*7,0)	(*s*6,0)
2	Simulator B	(*s*7,0)	(*s*7,-0.23)	(*s*6,0.33)	(*s*6,0)	(*s*7,0.03)	(*s*7,-0.23)
3	Simulator C	(*s*5,-0.23)	(*s*4,0.33)	(*s*4,0)	(*s*4,-0.23)	(*s*4,0.33)	(*s*5,-0.23)
4	Simulator D	(*s*5,0)	(*s*6,-0.23)	(*s*5,0.33)	(*s*5,0.33)	(*s*5,0.33)	(*s*5,0)
5	Simulator E	(*s*7,-0.23)	(*s*7,0.03)	(*s*7,-0.23)	(*s*5,-0.23)	(*s*6,0.33)	(*s*6,0.33)
6	Simulator F	(*s*7,0)	(*s*7,-0.23)	(*s*7,-0.23)	(*s*5,0)	(*s*6,0.33)	(*s*6,-0.23)
7	Simulator G	(*s*4,-0.23)	(*s*6,0)	(*s*6,0)	(*s*3,0)	(*s*4,-0.23)	(*s*3,0.33)
8	Simulator H	(*s*7,0)	(*s*6,0.33)	(*s*6,0)	(*s*5,0)	(*s*6,0)	(*s*6,0)
9	Simulator I	(*s*6,0)	(*s*6,-0.23)	(*s*6,0)	(*s*4,0.33)	(*s*5,0.33)	(*s*5,0)
10	Simulator J	(*s*8,-0.23)	(*s*7,0)	(*s*7,0.03)	(*s*6,-0.23)	(*s*7,-0.23)	(*s*6,-0.23)
11	Simulator K	(*s*5,0.33)	(*s*5,0.33)	(*s*6,-0.23)	(*s*4,0.33)	(*s*5,0.33)	(*s*5,-0.23)
12	Simulator L	(*s*6,0)	(*s*6,-0.23)	(*s*6,-0.23)	(*s*5,0.33)	(*s*5,0)	(*s*5,0)
13	Simulator M	(*s*6,0.25)	(*s*6,0)	(*s*6,0.25)	(*s*6,0.25)	(*s*6,0)	(*s*5,0)
14	Simulator N	(*s*6,-0.23)	(*s*6,0)	(*s*6,-0.23)	(*s*6,-0.23)	(*s*6,0)	(*s*5,0)
15	Simulator O	(*s*4,0.33)	(*s*5,-0.23)	(*s*4,0.33)	(*s*4,0)	(*s*4,0)	(*s*5,-0.23)

#### Step 5: Calculate the weighting of the evaluation indicators

The weighting calculation in this section is the same as that described in Section 4.2.1. Similarly, Expert Choice 2000 software was employed to calculate the weighting of each evaluation indicator. Regarding the importance dimension, the indicator “enhance overall training safety” was considered the most important, with a weighting value of 0.475, followed by “enhance teaching and training effectiveness” (0.160) and lastly “effectively reduce training cost” (0.149). Concerning the performance dimension, “subsequent expenses satisfy actual teaching needs” registered the heaviest weight at 0.096, followed by the “quantity of simulators satisfies actual teaching needs” (0.070) and “operating methods are the same as those of actual equipment” (0.051).

#### Step 6: Calculate and ranking evaluation performance

To further elucidate the ranking of the simulators in terms of their performance, the weights of each evaluation indicator obtained through Step 5 were multiplied with the ratings shown in Table 12. The summation of the results is shown in Table 13. Subsequently, the six indicators in Table 13 were summed and ranked according to their degrees of importance and performance, before the overall performance ranking for each simulator was performed (Table 14).

**Table 13:** Weight average of the simulator evaluation indicators.

			Importance			Performance	
	Benefit indicators	Effectively reduce training cost	Enhance overall training safety	Enhance teaching and training effectiveness	Subsequent expenses satisfy actual teaching needs	Operating methods are the same as those of actual equipment	Quantity of simulators satisfies actual teaching needs
1	Simulator A	(*s*1,0.28)	(*s*1,0.34)	(*s*1,0.41)	(*s*1,0.22)	(*s*1,0.34)	(*s*1,0.15)
2	Simulator B	(*s*1,0.34)	(*s*1,0.28)	(*s*1,0.22)	(*s*1,0.15)	(*s*1,0.41)	(*s*1,0.22)
3	Simulator C	(*s*1,-0.10)	(*s*1,-0.17)	(*s*1,-0.23)	(*s*1,-0.30)	(*s*1,-0.17)	(*s*1,0.-0.04)
4	Simulator D	(*s*1,-0.04)	(*s*1,0.09)	(*s*1,0.02)	(*s*1,0.02)	(*s*1,0.02)	(*s*1,-0.04)
5	Simulator E	(*s*1,0.28)	(*s*1,0.41)	(*s*1,0.28)	(*s*1,-0.10)	(*s*1,0.22)	(*s*1,0.22)
6	Simulator F	(*s*1,0.34)	(*s*1,0.28)	(*s*1,0.28)	(*s*1,-0.04)	(*s*1,0.22)	(*s*1,0.09)
7	Simulator G	(*s*1,-0.30)	(*s*1,0.15)	(*s*1,0.15)	(*s*1,-0.42)	(*s*1,-0.30)	(*s*1,-0.36)
8	Simulator H	(*s*1,0.34)	(*s*1,0.22)	(*s*1,0.15)	(*s*1,-0.04)	(*s*1,0.15)	(*s*1,0.15)
9	Simulator I	(*s*1,0.15)	(*s*1,0.09)	(*s*1,0.15)	(*s*1,-0.17)	(*s*1,0.02)	(*s*1,-0.04)
10	Simulator J	(*s*1,0.47)	(*s*1,0.34)	(*s*1,0.41)	(*s*1,0.09)	(*s*1,0.28)	(*s*1,0.09)
11	Simulator K	(*s*1,0.02)	(*s*1,0.02)	(*s*1,0.09)	(*s*1,-0.17)	(*s*1,0.02)	(*s*1,-0.10)
12	Simulator L	(*s*1,0.15)	(*s*1,0.09)	(*s*1,0.09)	(*s*1,0.02)	(*s*1,-0.04)	(*s*1,-0.04)
13	Simulator M	(*s*1,0.20)	(*s*1,0.15)	(*s*1,0.20)	(*s*1,0.20)	(*s*1,0.15)	(*s*1,-0.04)
14	Simulator N	(*s*1,0.09)	(*s*1,0.15)	(*s*1,0.09)	(*s*1,0.09)	(*s*1,0.15)	(*s*1,-0.04)
15	Simulator O	(*s*1,-0.17)	(*s*1,-0.10)	(*s*1,-0.17)	(*s*1,-0.23)	(*s*1,-0.23)	(*s*1,-0.04)

**Table 14:** Ranking of the simulator evaluation indicators.

		Importance		Performance		Total
	Benefit indicators	Aggregated value of weight average	Sorting of weight average	Aggregated value of weight average	Sorting of weight average	
1	Simulator A	(*s*4,0.03)	2	(*s*4,-0.29)	2	(*s*8,-0.26)
2	Simulator B	(*s*4,-0.16)	5	(*s*4,-0.22)	1	(*s*8,-0.38)
3	Simulator C	(*s*3,-0.50)	15	(*s*3,-0.50)	13	(*s*5,-0.01)
4	Simulator D	(*s*3,0.07)	12	(*s*3,0.01)	9	(*s*6,0.08)
5	Simulator E	(*s*4,-0.03)	3	(*s*3,0.33)	4	(*s*7,0.30)
6	Simulator F	(*s*4,-0.10)	4	(*s*3,0.26)	6	(*s*7,0.17)
7	Simulator G	(*s*3,0.01)	13	(*s*2,-0.08)	15	(*s*5,-0.07)
8	Simulator H	(*s*4,-0.29)	6	(*s*3,0.26)	6	(*s*7,-0.02)
9	Simulator I	(*s*3,0.39)	8	(*s*3,-0.18)	11	(*s*6,0.21)
10	Simulator J	(*s*4,0.22)	1	(*s*3,0.46)	3	(*s*7,-0.32)
11	Simulator K	(*s*3,0.14)	11	(*s*3,-0.25)	12	(*s*6,-0.11)
12	Simulator L	(*s*3,0.33)	9	(*s*3,-0.06)	10	(*s*6,0.27)
13	Simulator M	(*s*4,-0.45)	7	(*s*3,0.31)	5	(*s*7,-0.14)
14	Simulator N	(*s*3,0.33)	9	(*s*3,0.20)	8	(*s*7,-0.47)
15	Simulator O	(*s*3,-0.44)	14	(*s*3,-0.50)	14	(*s*5,0.06)

#### Step 7: Analyze simulator performance

**Fig 5:**
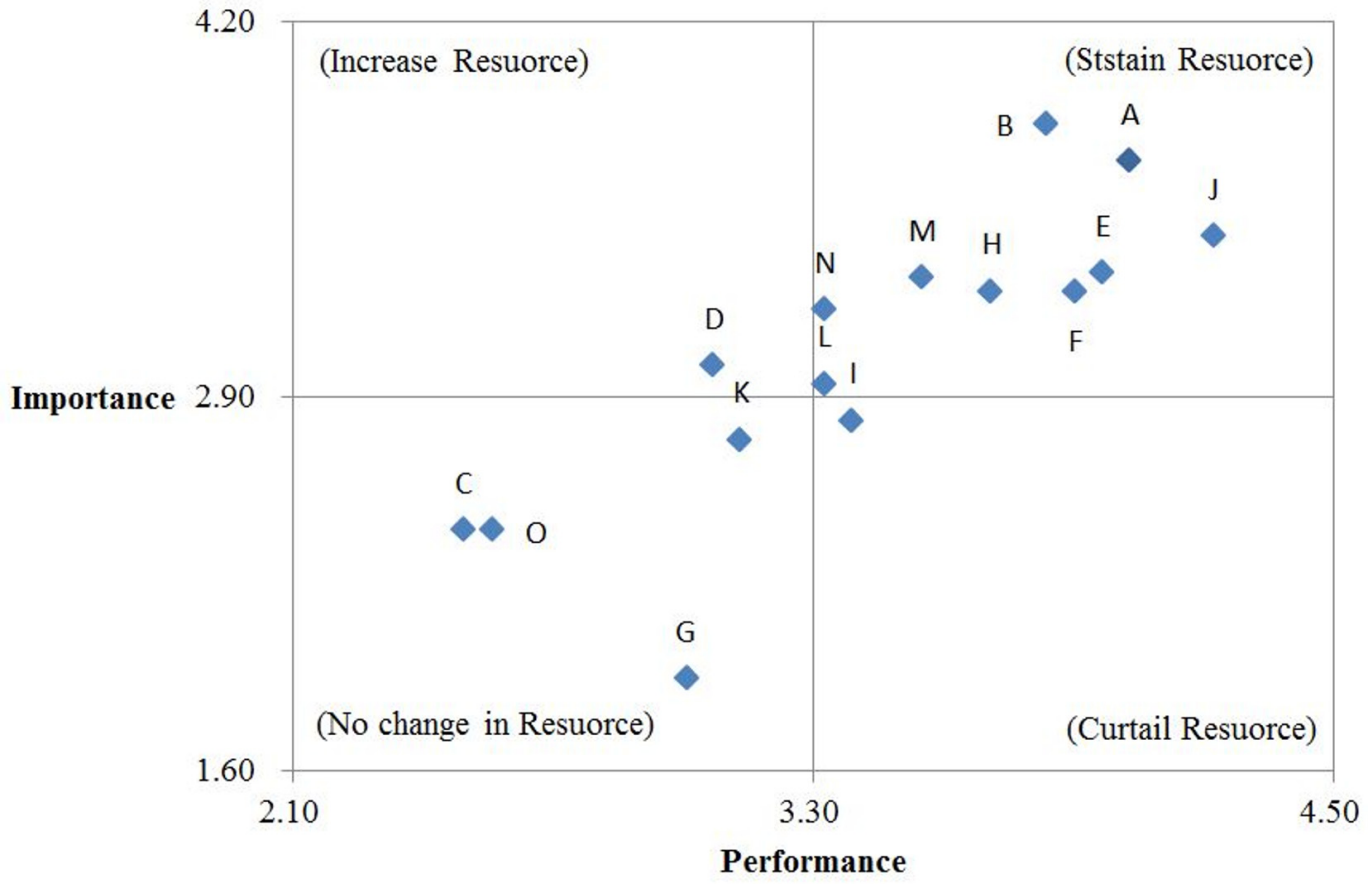
IPA diagram.

After Step 6 was completed, IPA was employed to analyze the performance of 15 simulators, using “performance” as the *X* axis on the two-dimensional quadrant diagram, and “importance” as the *Y* axis. The mean value of the importance and performance scores shown in Table 14 was calculated as (3.30, 2.90), which was used as the central point of the *X* and *Y* axes. Subsequently, the upper and lower limits of the *X* and *Y* axes were identified from among the distribution of numerical values, with *X* axis as (4.50, 2.10) and *Y* axis as (1.60, 4.20). Next, the importance and performance scores of the simulators in Table 14 were regarded as coordinates, and these coordinates were plotted onto the two-dimensional quadrant diagram (Fig 5). The 15 simulators in the four quadrants are analyzed and explained as follows.

1. Quadrant I (sustain resources): This quadrant comprised Simulators A, B, E, F, H, J, L, M, and N, suggesting that these simulators are highly important because of their function in enhancing training safety and effectiveness. In addition, they also exhibit excellent performance because their subsequent expenses and number of simulators available both satisfy actual teaching needs. Therefore, top priority in continuously sustaining these simulators is required to maintain training effectiveness.
2. Quadrant II (increase resources): This quadrant comprised Simulator D, suggesting that this simulator is important but its performance cannot be improved because of limited resources. In future, more resources should be invested into this simulator to enhance its performance.
3. Quadrant III (no change in resources): This quadrant comprised Simulators C, G, K, and O, meaning that these simulators exhibit low importance and low performance. When resources are limited, management units could reflect and contemplate on the budget allocation for these types of simulators in order to improve the overall benefits of the budget expended on these simulators.
4. Quadrant IV (curtail resources): This quadrant comprised Simulator I, indicating that this simulator was recognized for its performance. However, because this simulator is not as important as the simulators in Quadrants 1 and 2, overinvestment in this simulator is not required. Resources should be utilized on other simulators that exhibit high importance and performance to avoid wasting resources and to maximize the overall benefits of training simulators.

### Comparisons and discussion

#### The reason for using the AHP, IPA and Soft set

Today, military forces worldwide are already adopting simulators to train their military armies. This approach lowers training costs and prevents unnecessary accidents and casualties. However, because of the adjusted reduction in national defense budgets, worldwide governments must evaluate the benefits of their available simulators when usable resources are limited. Thus, the limited resources can be invested in simulators that yield better investment returns and training effectiveness. However, because the development times for simulators differ, and each simulator differs in terms of its usage purpose, these discrepancies indirectly cause inconsistent operating functions, which make it difficult to evaluate the overall benefits of multiple simulators with a single standard. AHP is a method that decomposes a complex problem hierarchically from top to bottom to identify the influencing factors of the problem. Subsequently, experts are invited to compare the importance of a pair of qualitative influencing factors and subjectively rate them on a questionnaire to quantify these factors. Thereafter, weighting calculation is performed to rank these factors in terms of their importance, thereby determining the most optimal solution and simplifying the complex decision-making process.

During the questionnaire survey process, certain respondents complete only the questions they understand, are familiar with, or are interested in. Thus, some questionnaires were incomplete, which are deemed as invalid questionnaires according to traditional questionnaire analysis methods. Consequently, some crucial information might be neglected, causing the conclusion drawn from the questionnaire results to differ substantially from real-life situations. Nevertheless, soft set theory can be applied to supplement and fully account for the missing information. This approach avoids the loss of valuable information, lowers the bias caused by the use of inaccurate raw data during the calculation process, and generates results that are authentic and more accurately reflect the conditions in real life.

Furthermore, past studies on performance evaluation have largely focused on obtaining a single solution to a problem such as improving the performance of simulators or how to reduce training costs. In addition, these studies typically compare the advantages and disadvantages of the targets or items under evaluation by ranking them, thus allowing administrators to understand only the priority order of the evaluated items from the rankings, instead of determining the relative relationship between the items. IPA simultaneously evaluates the importance and performance of various items, using two-dimensional graphs to classify the evaluation results into four quadrants. Next, this method analyzes the phenomena and implications of the evaluated items in each quadrant, providing administrators with a reference to manage the items and identify the best strategy for solving their problems.

In summary, AHP considers both qualitative and quantitative problems, performs hierarchical and structural analyses the most optimal solution to the problem of interest. On the other hand, Chang et al. [64] proposed method that integrates AHP, IPA, and 2-tuple fuzzy linguistic representation model, and use solving characteristic of method to evaluate the benefit of military simulation training systems effectively, it not only accurately predicts the priority of simulators, but also provides the correct information for managers, and further guide the decision-making process. However, these two research methods failed to consider the crucial information might be contained within these invalid questionnaires, and thus they might have neglected some of the information provided by the experts. As a result, the analysis results might not truly reflect the real conditions. This study proposed 2-tuple AHP method, it not only integrates AHP, IPA, and 2-tuple fuzzy linguistic representation model to evaluate the overall performance of a training simulator system, but also use soft set to supplement incomplete information of questionnaires. It can consider full information to avoid the bias in the numerical values during the calculation process, which makes result more realistic. Due to 2-tuple AHP method is more general research method; therefore, it has fewer limitations than the AHP and Chang et al.’s method [64]. The main differences in special attributes among AHP, Chang et al.’s method [64] and 2-tuple AHP method are summarized in Table 15.

**Table 15:** The difference among three kinds of research methods.

	Solving characteristic		
Research method	Supplement incomplete information	Consider valuable information fully	Evaluates the performance by two-dimensional graphs
AHP	No	No	No
Chang et al.’s method [64]	No	Yes	Yes
Proposed method	Yes	Yes	Yes

#### Comparison of the results of the research methods used in this study

This proposed 2-tuple AHP method that integrates soft-set, 2-tuple AHP, IPA to evaluate the importance and performance of 15 training simulators. To facilitate accurate result presentation and comparison, the results in Tables 8, 11 and 14 obtained using the three methods respectively, were summarized as shown in Table 16.

**Table 16:** Comparison of the results of AHP and the methods by Chang et al. [64] and this study.

Item	Simulators	Aggregated value by using AHP	Aggregated value by using Chang et al.’s [64] method	Aggregated value by using this study’s proposed method	Ranking by using AHP	Ranking by using Chang et al.’s [64] method	Ranking by using this study’s proposed method
1	Simulator A	6.79	(*s*6,0.40)	(*s*8,-0.26)	2	3	1
2	Simulator B	6.21	(*s*6,0.21)	(*s*8,-0.38)	6	5	3
3	Simulator C	4.60	(*s*4,0.21)	(*s*5,-0.01)	14	15	14
4	Simulator D	5.22	(*s*5,0)	(*s*6,0.08)	13	12	11
5	Simulator E	6.69	(*s*7,-0.49)	(*s*7,0.30)	3	2	4
6	Simulator F	6.63	(*s*6,0.21)	(*s*7,0.17)	5	5	5
7	Simulator G	5.25	(*s*5,-0.17)	(*s*5,-0.07)	11	13	15
8	Simulator H	6.69	(*s*6,0.34)	(*s*7,-0.02)	3	4	6
9	Simulator I	6.00	(*s*6,-0.26)	(*s*6,0.21)	7	7	10
10	Simulator J	7.20	(*s*7,-0.06)	(*s*7,-0.32)	1	1	2
11	Simulator K	5.69	(*s*5,0.30)	(*s*6,-0.11)	9	10	12
12	Simulator L	5.25	(*s*5,0.20)	(*s*6,0.27)	11	11	9
13	Simulator M	5.82	(*s*6,-0.26)	(*s*7,-0.14)	8	7	7
14	Simulator N	5.69	(*s*5,0.31)	(*s*7,-0.47)	9	9	8
15	Simulator O	4.60	(*s*4,0.34)	(*s*5,0.06)	14	14	13

In the past, when survey questionnaires are being analyzed statistically, questionnaires with missing information are typically discarded as invalid questionnaires; this approach generally neglects some important information. The AHP and the method proposed by Chang et al. [64] summarize and compute data according to traditional questionnaire analysis methods, which is why they overlooked some of the information provided by experts, obtaining results that differ from actual situations. Nevertheless, the present study attempted to retain crucial questionnaire information by using soft set theory to supplement missing information so that information can be fully considered and extensively applied. Thus, all questionnaire information can be presented authentically.

According to Tables 8, 11 and 14, the traditional AHP method showed that Simulators E and H both received a rating of “7” for their ability to effectively reduce training costs. This method not only failed to effectively distinguish the pros and cons of these two simulators in this regard, but also led to a series of bias in the calculation process due to inaccurate raw data. The method proposed in the present study and that developed by Chang et al. [60] applied the 2-tuple fuzzy linguistic representation model; during the questionnaire collection process, comprehensive rating values can be obtained. The proposed method of this study obtained a rating of (s7, -0.5) and (s7, 0) for Simulators E and H, respectively, whereas Chang et al.’s method [64] yielded a rating of (s7, -0.23) and (s7, 0), respectively. This shows that both methods did not neglect certain information presented in the raw questionnaire data, which would otherwise engender continuous bias in the calculation process. Table 16 reveals the AHP calculation results, indicating that Simulators E and H were ranked third place among all of the 15 simulators, whereas Simulators K and N were ranked ninth, Simulators G and L ranked eleventh, and Simulators C and O were ranked fourteenth. By contrast, although Chang et al.’s approach [64] employed the 2-tuple fuzzy linguistic model, it did not incorporate missing questionnaire information into consideration. Consequently, Chang et al.’s approach [64] revealed that Simulators B and F were ranked fifth whereas Simulators I and M were ranked seventh. This result did not immediately elucidate the strength and weakness of the simulators, which would impede the process of resource allocation. Nevertheless, the method proposed in the present study presented no repeated rankings, effectively ranked simulator performance, and efficiently provided an effective reference for resource allocation.

Finally, Table 16 shows that although the three methods effectively ranked the 15 simulators, a considerable time was required to analyze the ranking, strengths, and weaknesses of various simulators. The methods proposed in this study and by Chang et al. [64] employed IPA method to display the performance and importance values of the 15 simulators on a two-dimensional quadrant diagram. This diagram clearly reveals the quadrant to which simulators belong. Thus, administrators could easily ascertain how to manage simulators, properly maintain them, and allocate simulator budgets effectively according to the meaning and characteristics representative of the quadrants.

## Conclusions

The advancement of high-tech applications and the arrival of the information age have led to constant changes to the forms of modern warfare. To attain the goals of military mission training, governments around the globe are also prompted to train military armies by using simulation training systems instead of having soldiers use the actual military equipment in training. However, various training simulators have not yet been evaluated comprehensively, and this is difficult to achieve because the establishment time, functions, environment, and the capabilities of administrators and operators associated with simulator systems vary considerably and because questionnaire surveys occasionally yield incomplete data. To address this problem and avoid wasting training resources, this study proposed an evaluation method that integrates 2-tuple AHP, soft set theory, and IPA. The proposed method was used to evaluate the performance of 15 training simulator systems. As described in Section 4, the verification of the numerical values revealed that the proposed method has four advantages:

1. It does not lose important information provided by experts.
2. It fully consider incomplete information.
3. It effectively reduces the chance of obtaining repeated ranking results.
4. It generates two-dimensional graph that presents information clearly.

The proposed 2-tuple AHP method verified that the characteristics of various research methods in solving problems can be effectively applied to accurately analyze expert-provided information and rank the performance of various simulators. Thus, graphical information is provided to simulator operators so that they can ascertain the relationship of the evaluated simulators regarding their importance and performance. Moreover, when simulator investment and maintenance budgets are limited, simulator operators could use the results obtained from the proposed method to appropriately and effectively allocate resources, properly manage training simulation systems, and thereby maximize the overall training effectiveness.
